# Supplementary material for: Generation of pluripotent stem cell-derived mouse kidneys in Sall1-targeted anephric rats
Source: Nat Commun. 2019 Feb 5;10:451. doi: 10.1038/s41467-019-08394-9 (PMC6363802; doi:10.1038/s41467-019-08394-9)
Supplement: Supplementary file 1 — Supplementary Information [file 41467_2019_8394_MOESM1_ESM.pdf]

Supplementary Information

Generation of pluripotent stem cell-derived mouse kidneys in *Sall1*-targeted anephric rats

Teppei Goto et al.

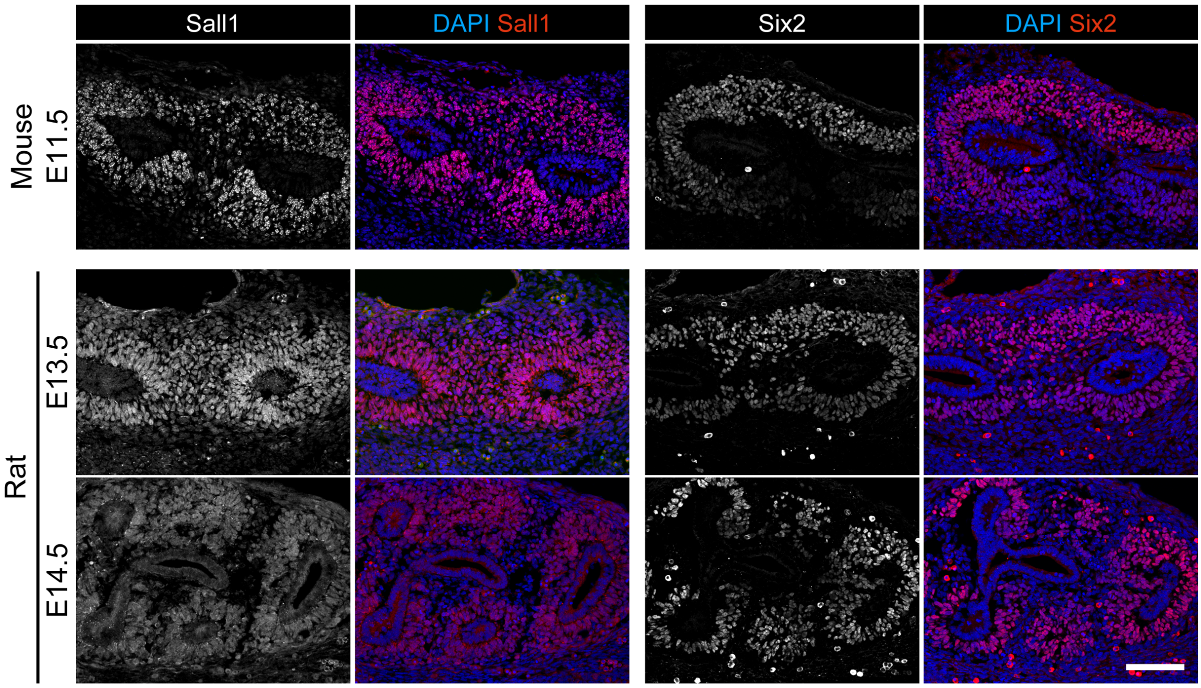

Supplementary Figure 1. Developmental characterization of metanephric mesenchyme in mouse and rat.

Sall1 (left panels) and Six2 (right panels) expression in mouse E11.5, and rat E13.5 and E14.5, respectively. Scale bar: 100 μm.

a

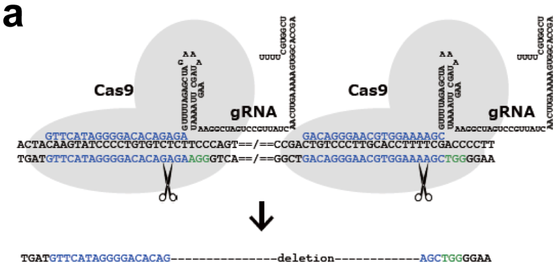

b

	E21.5 fetuses	Full-term offspring
Zygotes injected	228	
survived	184 (81%)	
cleaved	156 (85%)	
transferred	156	28
Fetuses or offspring	8 (5%)	4 (14%)
Genotype +/+	2 [#5, #10]	1 [#4]
Genotype +/mut	3 [#6, #9, #12]	3 [#1, #2, #3]
Genotype mut/mut	3 [#7, #8, #11]	0

c

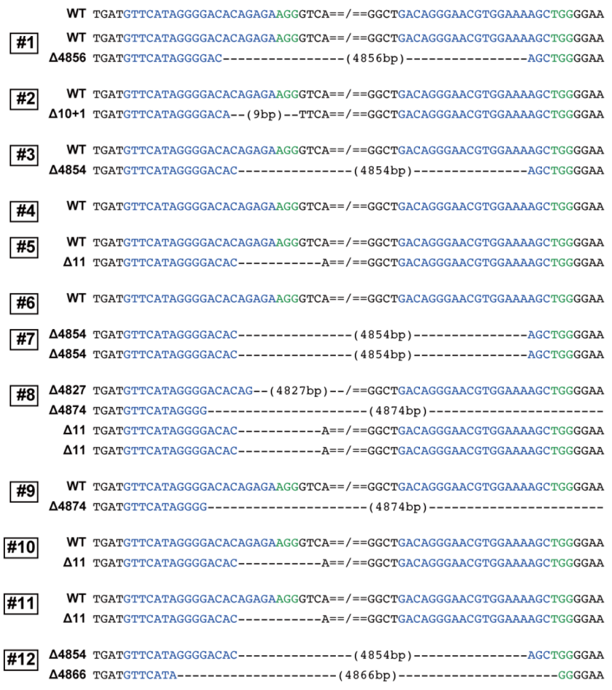

Supplementary Figure 2. Generation of *Sall1*$^{mut/mut}$ rat lines via the CRISPR/Cas9 system.

(**a**) Two guide RNA sequences targeting for the second and third exons of *Sall1* were cloned into px330 vector expressing Cas9 and single-guide RNA. (**b**) A mixture of 2 px330 vectors were microinjected into pronuclear-stage rat zygotes. Twelve chimeric fetal or newborn rats were generated, and three homozygotic mutants (#7, #8 and #11) had an anephric phenotype. (**c**) Sequencing of CRISPR#1–#12 rats suggested that the large deletion in both alleles of the *Sall1* target region (≈ 4.8 kb) was associated with anephric phenotype. A large deletion in the single *Sall1* allele was used to establish the mutant rat line CRISPR#1. Blue and green letters indicate target and PAM sequences, respectively.

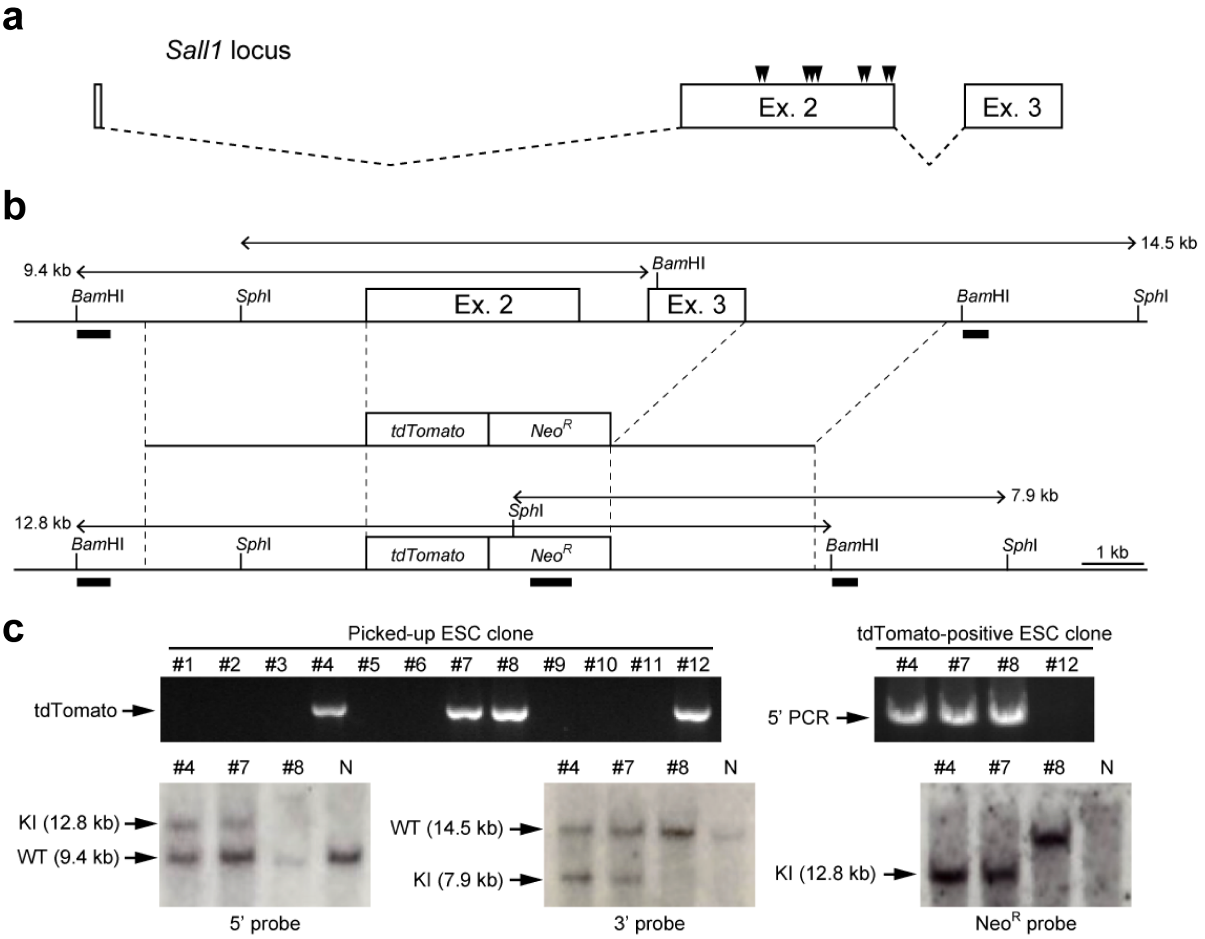

d

Rat ESC clone #4

Blastocysts injected	Offspring born	Chimeric rats	ID	G1 offspring born	G1 offspring with black coat	G1 offspring with tdTomato
48	17	13 (76%)	#4-1	36	18	8
			#4-2	35	0	0
			#4-3	38	14	7
			#4-4	40	12	0
			#4-5	43	0	0
			#4-6	37	32	11
			#4-7	25	5	1

Supplementary Figure 3. Generation of *Sall1*$^{mut/mut}$ rat lines via *tdTomato*-knock-in by conventional homologous recombination in rat ESCs.

(**a**) Structure of rat *Sall1* locus. Arrowheads represent DNA-binding domains. (**b**) Design of the targeting vector to replace the second and third exons of *Sall1* with *tdTomato* and *Neo*^{R} cassette. (**c**) PCR screening of ESC clones (#1–12) to detect

tdTomato (upper left) and 5’ recombination (upper right). Southern blot analysis confirmed ESC clones #4 and #7 were successfully targeted. *Bam*HI-digests hybridized with 5' probe (bottom left), *Sph*I-digests hybridized with 3' probe (bottom center), and *Bam*HI-digests hybridized with Neo probe (bottom right). N: negative control. (**d**) Germline transmission of knocked-in tdTomato gene via conventional progeny test. Black-coated and tdTomato-positive G1 offspring were used to establish the HR#4 rat line.

a

	Blastocyst injected	Living fetuses	Chimeric fetuses	Genotype				Kidney complemented
				Wildtype	Hetero	Homo	Not determined	
HR#4	48	27 (56%)	11 (41%)	3	6	2	0	2 (100%)
CRISPR#1	32	25 (78%)	13 (52%)	5	5	2	1	1 (50%)

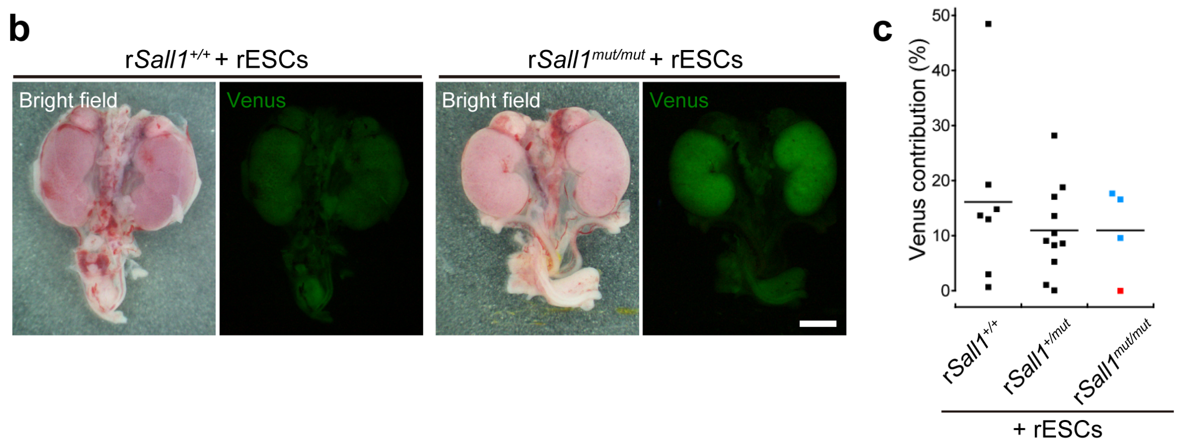

d

	Blastocyst injected	Living fetuses	Chimeric fetuses	Genotype			Kidney complemented
				Wildtype	Hetero	Homo	
CRISPR#1	90	50 (61%)	15 (27%)	4	10	1	1 (100%)

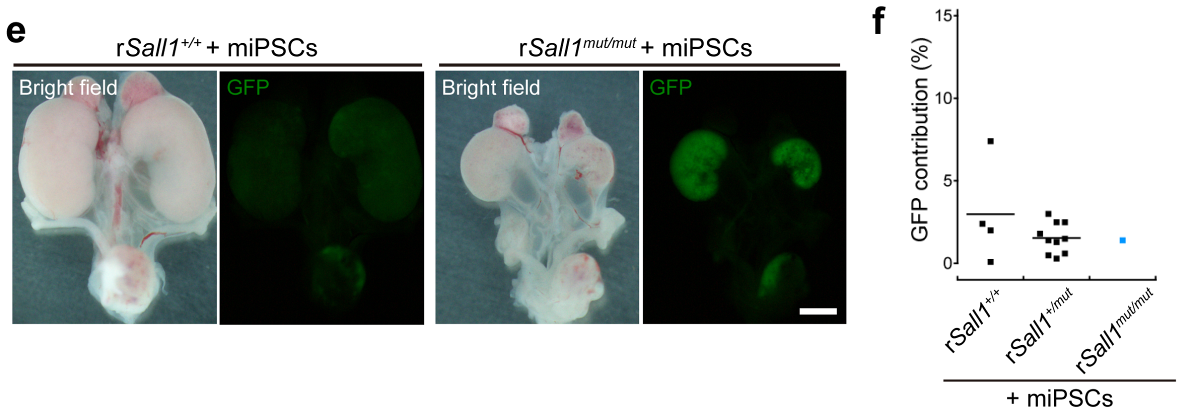

Supplementary Figure 4. Generation of kidney by injection of rat ESCs or mouse iPSCs into *Sall1*$^{mut/mut}$ rat blastocysts.

(**a**) Generation of allogenic chimeras by injection of Venus-labeled rat ESCs into rat blastocysts obtained from intercrossing of *Sall1*$^{+/mut}$ rats. (**b**) Venus fluorescence derived from rat ESCs in kidneys of *Sall1*$^{+/mut}$ and *Sall1*$^{mut/mut}$ chimeras. Scale bar: 2 mm. (**c**) Variablity of chimerism in splenic lymphocytes. Bars represent the mean percentage. Blue and red boxes for *Sall1*$^{mut/mut}$ genotype represent nephric and anephric phenotype, respectively. (**d**) Generation of interspecific chimeras by injection of GFP-labeled mouse iPSCs into rat blastocysts obtained from

intercrossing of *Sall1*$^{+/mut}$ rats. (**e**) GFP fluorescence derived from mouse iPSCs in kidneys of *Sall1*$^{+/mut}$ and *Sall1*$^{mut/mut}$ chimeras. Scale bar: 2 mm. (**f**) Variability of chimerism in splenic lymphocytes. Bars represent the mean percentage. Blue boxes for *Sall1*$^{mut/mut}$ genotype represent chimera with complemented kidney pair. All data were obtained from 2 independent experiments. Source data are provided as a Source Data file.

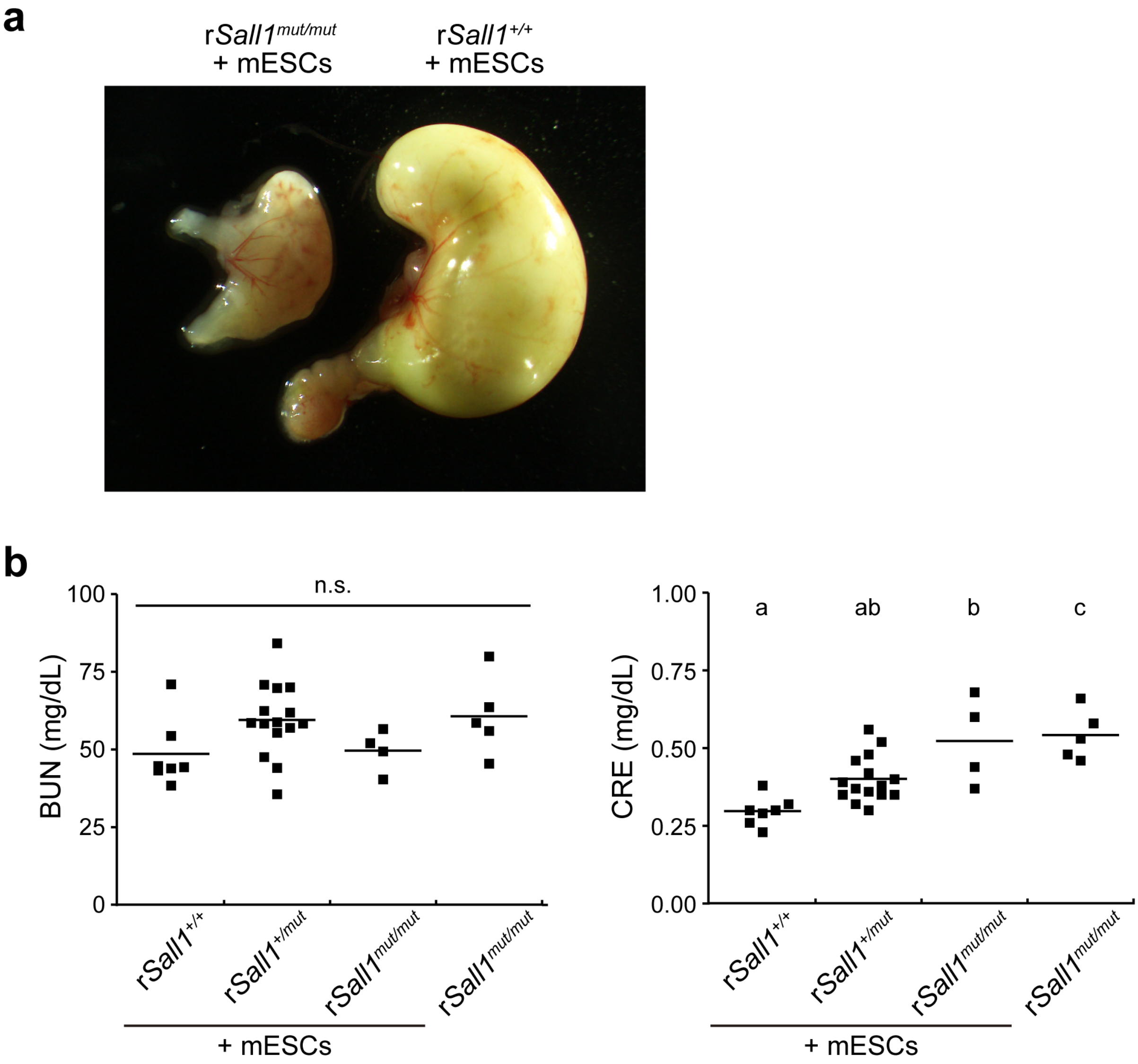

Supplementary Figure 5. Postnatal characterization of *Sall1*$^{mut/mut}$ chimeric rats with mouse ESC derived kidney.

(**a**) Representative photomicrograph of a shrunken (*Sall1*$^{mut/mut}$: left side) and a normal (*Sall1*$^{+/+}$: right side) stomach at neonatal stage. (**b**) The serum levels of BUN and CRE from *Sall1*$^{+/+}$ chimeras, *Sall1*$^{+/mut}$ chimeras, *Sall1*$^{mut/mut}$ chimeras with mouse PSC-derived kidneys, and *Sall1*$^{mut/mut}$ rats without kidneys at neonatal stage. Bars represent the mean percentage. All data were obtained from 3 independent experiments. Different letters (a, b, and c) indicate statistically significant differences ($p < 0.05$) based on a one-way ANOVA followed by Tukey's HSD post hoc test. n.s.:

Not statistically significant by one-way ANOVA. Source data are provided as a Source Data file.

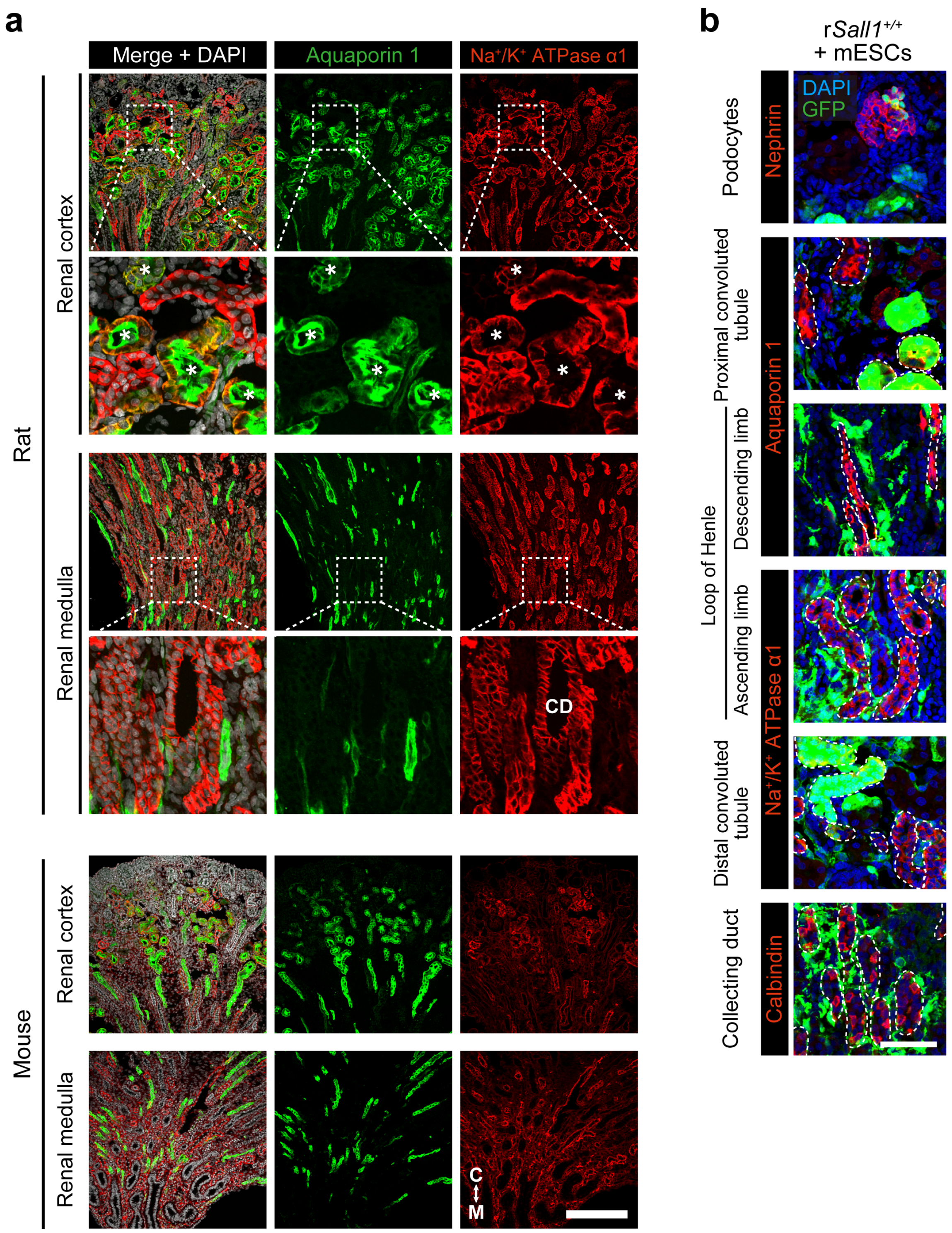

Supplementary Figure 6. Immunohistochemical staining of neonatal kidneys.

(**a**) Images of renal cortex and medulla in rat and mouse. Aquaporin 1 and Na^+/K^+ ATPase α-1-double positive cells (see asterisks in higher magnification images) show proximal convoluted tubules. Na^+/K^+ ATPase α-1-single positive cells show distal

convoluted tubules in renal cortex. Na^+/K^+ ATPase α-1-weak positive cells show collecting duct in renal medulla of rat. C <-> M represents cortex and medulla, respectively. The results are summarized in **Supplementary Table 1**. Scale bar: 200 µm. (**b**) Immunohistochemical staining of neonatal kidneys in *Sall1*$^{+/+}$ chimeric rat with mouse ESCs. Neonatal kidneys for GFP (green) with markers for each renal component (red); Nephrin for Podocytes, Aquaporin 1 for proximal convoluted tubule in the renal cortex and thin descending limb/Henle's loop in the renal medulla, Na^+/K^+ ATPase α-1 for thick ascending limb in the renal medulla and distal convoluted tubule. Calbindin for collecting tubule in the renal medulla. The nuclei were stained with DAPI (blue). Scale bar: 50 µm.

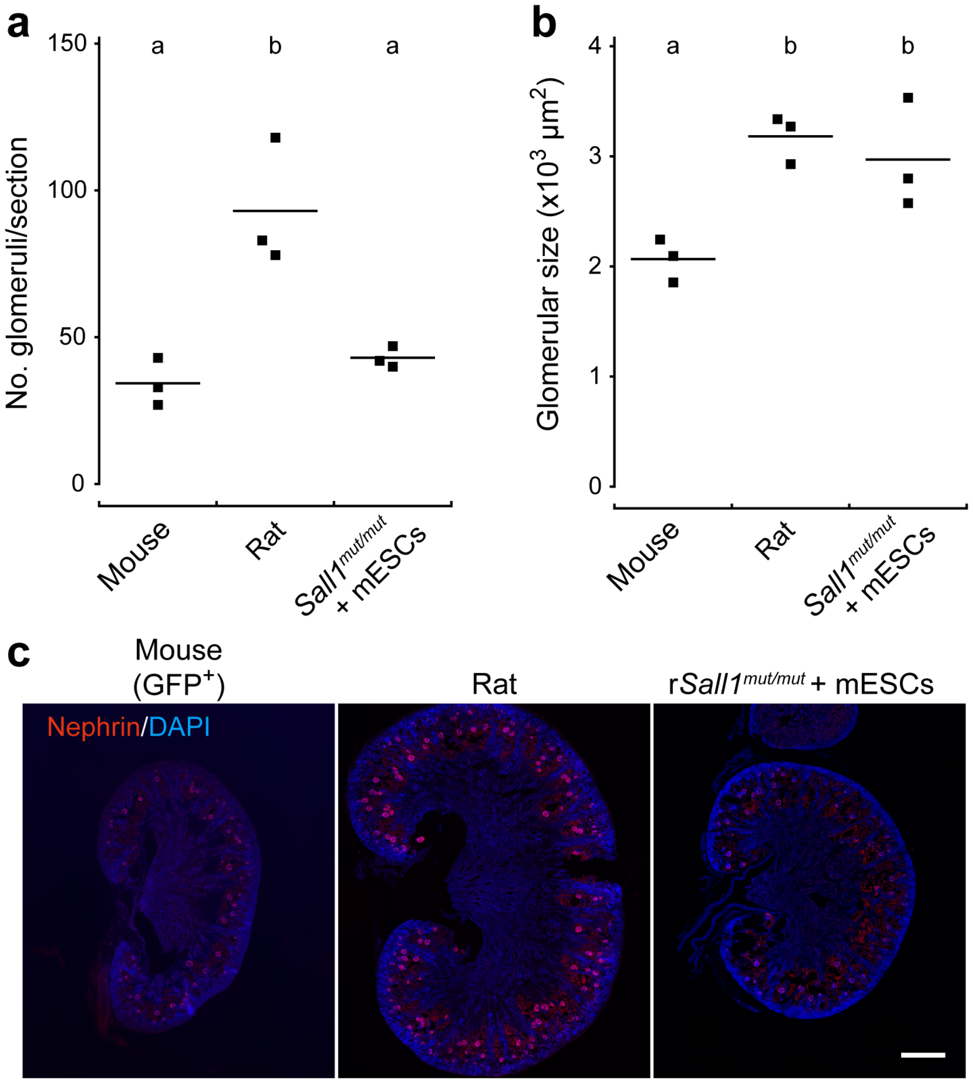

Supplementary Figure 7. Glomerular number and size of *Sall1*$^{mut/mut}$ chimeric rats compared with those of control mice and rats.

(**a**) The number of glomeruli counted in a maximum longitudinal and transverse kidney section of *Sall1*$^{mut/mut}$ chimeras, control mice and rats. Bars represent the mean number. (**b**) The mean size of glomeruli of *Sall1*$^{mut/mut}$ chimeras, control mice and rats. Bars represent the mean size. Different letters (a and b) indicate statistically significant differences ($p < 0.05$) based on a one-way ANOVA followed by Tukey's HSD post hoc test. (**c**) Representative image of the maximum longitudinal and transverse section of entire kidney. Nephrin (red) and DAPI (blue) visualized podocytes in glomeruli and cell nuclei, respectively. Scale bar: 500 µm. Source data are provided as a Source Data file.

Supplementary Table 1. Renal cell lineage marker for neonatal kidney of rat and mouse

	Cell lineage	Rat	Mouse
Cortex	podocyte	Podocin, Nephrin	Nephrin
	proximal tube	Aquaporin 1, Na^+/K^+ ATPase α1 (W)	Aquaporin 1, Na^+/K^+ ATPase α1 (W)
	distal tube	Na^+/K^+ ATPase α1 (S)	Na^+/K^+ ATPase α1 (S)
Medulla	TDL	Aquaporin 1	Aquaporin 1
	loop of Henle	Aquaporin 1	Aquaporin 1
	TAL	Na^+/K^+ ATPase α1 (S)	Na^+/K^+ ATPase α1 (S)
	Collecting duct	Calbindin<br>Na^+/K^+ ATPase α1 (W)	-

TDL: Thin descending limb of Henle's loop, TAL: Thick ascending limb of Henle's loop.
(W): weak signals, (S): strong signals.

Supplementary Table 2. Primer sequences for screening *Sall1*-targeted ESC clones and genotyping

	Forward primer (5'->3')	Reverse primer (5'->3')	Product size (bp)
PCR screening			
tdTomato	GCGAGGAGGTCATCAAAGAG	GATGACGGCCATGTTGTTGT	740
5’ PCR	GTCCTTCAGTGTCTTCCCGG	TGGATGTGGAATGTGTGCGA	7,092
Southern blotting			
5' probe	AGTGTTCAATTTGATTCATCCACAG	ACCAGGCTCTACCAGATTTAGTTTC	537
3' probe	GCAGTGGGTGATATTTTAACAGCTA	TGTCTGATCTTGAGGAAAGTAGAGG	409
Neo^R probe	GAACAAGATGGATTGCACGCAGGTTCTCCG	GTAGCCAACGCTATGTCCTGATAG	668
Indel detection			
F1-R1	TGGCCCTTTCTGTCATTTTC	TGCTGTTGATCACCGAGAAG	568 (Target 1)
F2-R2	TGGCCCCATGACATTTCTAGG	CGGGCGGCATTTTGAGTAGA	411 (Target 2)
F1-R3	TGGCCCTTTCTGTCATTTTC	CTCTGGCAGCTTTAGCTCGT	5,079 (Target 1-2)
Conventional genotyping			
F1-R1	TGGCCCTTTCTGTCATTTTC	TGCTGTTGATCACCGAGAAG	568 (WT allele)
F1-R3	TGGCCCTTTCTGTCATTTTC	CTCTGGCAGCTTTAGCTCGT	227 (*Sall1*-KO allele)
F1-R4	TGGCCCTTTCTGTCATTTTC	GATGACGGCCATGTTGTTGT	855 (*tdT*-KI allele)

WT: wildtype, *Sall1*-KO: *Sall1*-knockout, *tdT*-KI: *tdTomato*-knockin.
