# Supplementary material for: Generation of pluripotent stem cell-derived mouse kidneys in Sall1-targeted anephric rats
Source: Nat Commun. 2019 Feb 5;10:451. doi: 10.1038/s41467-019-08394-9 (PMC6363802; doi:10.1038/s41467-019-08394-9)
Supplement: Supplementary file 3 — Reporting Summary [file 41467_2019_8394_MOESM3_ESM.pdf]

nature research

Corresponding author(s): Masumi Hirabayashi

Last updated by author(s): 2018/12/21

Reporting Summary

Nature Research wishes to improve the reproducibility of the work that we publish. This form provides structure for consistency and transparency in reporting. For further information on Nature Research policies, see Authors & Referees and the Editorial Policy Checklist.

Statistics

For all statistical analyses, confirm that the following items are present in the figure legend, table legend, main text, or Methods section.

n/a	Confirmed	
☐	☒	The exact sample size (*n*) for each experimental group/condition, given as a discrete number and unit of measurement
☐	☒	A statement on whether measurements were taken from distinct samples or whether the same sample was measured repeatedly
☐	☒	The statistical test(s) used AND whether they are one- or two-sided<br>*Only common tests should be described solely by name; describe more complex techniques in the Methods section.*
☒	☐	A description of all covariates tested
☐	☒	A description of any assumptions or corrections, such as tests of normality and adjustment for multiple comparisons
☐	☒	A full description of the statistical parameters including central tendency (e.g. means) or other basic estimates (e.g. regression coefficient) AND variation (e.g. standard deviation) or associated estimates of uncertainty (e.g. confidence intervals)
☐	☒	For null hypothesis testing, the test statistic (e.g. *F*, *t*, *r*) with confidence intervals, effect sizes, degrees of freedom and *P* value noted<br>*Give P values as exact values whenever suitable.*
☒	☐	For Bayesian analysis, information on the choice of priors and Markov chain Monte Carlo settings
☒	☐	For hierarchical and complex designs, identification of the appropriate level for tests and full reporting of outcomes
☒	☐	Estimates of effect sizes (e.g. Cohen's *d*, Pearson's *r*), indicating how they were calculated

Our web collection on statistics for biologists contains articles on many of the points above.

Software and code

Policy information about availability of computer code

Data collection	SH-800 for FACS data, and Excel.
Data analysis	jS-STAR for statistical analysis, SH-800 for FACS analysis, Fiji for quantification of images.

For manuscripts utilizing custom algorithms or software that are central to the research but not yet described in published literature, software must be made available to editors/reviewers. We strongly encourage code deposition in a community repository (e.g. GitHub). See the Nature Research guidelines for submitting code & software for further information.

Data

Policy information about availability of data

All manuscripts must include a data availability statement. This statement should provide the following information, where applicable:

- Accession codes, unique identifiers, or web links for publicly available datasets
- A list of figures that have associated raw data
- A description of any restrictions on data availability

The data that support the findings of this study are available from the corresponding author upon reasonable request.

Field-specific reporting

Please select the one below that is the best fit for your research. If you are not sure, read the appropriate sections before making your selection.

☒ Life sciences ☐ Behavioural & social sciences ☐ Ecological, evolutionary & environmental sciences

For a reference copy of the document with all sections, see nature.com/documents/nr-reporting-summary-flat.pdf

Life sciences study design

All studies must disclose on these points even when the disclosure is negative.

Sample size	Sample size was depended on developmental ability and genotype of interspecific chimeras.
Data exclusions	No data were excluded from the analyses.
Replication	All data exclude generation of Sall1 knockin/knockout animals were obtained multiple experiments. Described in figure legends.
Randomization	This experimental design does not contain a comparison between groups that needs to consider randomization.
Blinding	This experimental design does not contain a comparison between groups that needs to consider blinding.

Reporting for specific materials, systems and methods

We require information from authors about some types of materials, experimental systems and methods used in many studies. Here, indicate whether each material, system or method listed is relevant to your study. If you are not sure if a list item applies to your research, read the appropriate section before selecting a response.

Materials & experimental systems

n/a	Involved in the study	
☐	☒	Antibodies
☐	☒	Eukaryotic cell lines
☒	☐	Palaeontology
☐	☒	Animals and other organisms
☒	☐	Human research participants
☒	☐	Clinical data

Methods

n/a	Involved in the study	
☒	☐	ChIP-seq
☐	☒	Flow cytometry
☒	☐	MRI-based neuroimaging

Antibodies

Antibodies used	The primary antibodies for immunohistochemistry used in this study as follows: anit-Sall1 polyclonal antibody (rabbit IgG, ab31526, Abcam plc.), anit-Six2 polyclonal antibody (rabbit IgG, 11562-1-AP, Proteintech Group, Inc.), anti-GFP polyclonal antibody (Chick IgY, ab13970, Abcam), anti-DsRed polyclonal antibody (rabbit IgG, 1:200 dilution; 632496, Takara Bio Inc.), anti-Nephrin polyclonal antibody (goat IgG, AF3159-SP, R&D systems Inc.), anti-Podocin polyclonal antibody (rabbit IgG, P0372, Sigma-Aldrich), anti-Calbindin polyclonal antibody (mouse monoclonal IgG, 1:200 dilution; ab82812, Abcam), anti-Aquaporin 1 polyclonal antibody (rabbit IgG, AB2219, Merck Millipore), anti-Na+/K+ ATPase α-1 monoclonal antibody (mouse IgG, 05-369, Merck Millipore) and anti-CD31 polyclonal antibody (rabbit IgG, ab28364, Abcam). The secondary antibodies used in this study as follows: Alexa488, Alexa546 or Alexa647 (Thermo Fisher Scientific Inc.).
Validation	All primary antibodies were validated by the supplier.<br>According to previous literatures, we validated that all primary antibodies could be detected expected tissues in our lab by immunohistochemistry on embryonic or neonatal kidney section from Crlj:WI and C57BL/6NCrSlc, as controls. In addition, the anti-Sall1 and anti-DsRed antibodies were validated in our lab on embryonic kidney section from Sall1-tdTomato homozygous and heterozygous rats.

Eukaryotic cell lines

Policy information about cell lines

Cell line source(s)	Rat ES cells (WDB/Nips-ES1/Nips, RGD ID: 10054010; Crlj:WI-ES1/Nips, RGD ID: 10053737; WDB-Rosa26em1(RT2)Nips-ES2/Nips, RGD ID: 10054032) were established in our laboratory, as previously reported (Hirabayashi et al. J Reprod Dev 2014). Mouse PS cells (SGE2 and GT3.2) were established in Hiro Nakauchi laboratory, as previously reported (Usui et al., Am J Pathol, 2012 and Yamaguchi et al., Nature, 2017).<br>Neo-MEFs (KBL9284100) were purchased form Oriental Yeast Co., Ltd. Tokyo, Japan.
Authentication	Rat ES cells and mouse PS cells were authenticated by fluorescence in colonies. Neo-MEFs were received directly from the supplier (Oriental Yeast Co., Ltd.) and thus not further authenticated.
Mycoplasma contamination	All cell lines used in this study were tested negative for mycoplasma contamination in previous papers (Yamaguchi et al. Nature 2017, Hirabayashi et al. J Reprod Dev 2014) and not further tested in this study.
Commonly misidentified lines<br>(See ICLAC register)	No commonly misidentified cell lines were used.

Animals and other organisms

Policy information about studies involving animals; ARRIVE guidelines recommended for reporting animal research

Laboratory animals	Crlj:WI, Sall1+/+, Sall1mut/mut, Sall1+/mut, Sall1tdtomato/tdtomato, and Sall1+/tdtomato rats.<br>C57BL/6NCrSlc mice and C57BL/6N-Tg (CAG-EGFP) mice.
Wild animals	No wild animals were used in this study.
Field-collected samples	No field-collected samples were used in this study.
Ethics oversight	All procedures for animal experimentation were reviewed and approved by the Animal Care and Use Committee of the National Institute for Physiological Sciences.

Note that full information on the approval of the study protocol must also be provided in the manuscript.

Flow Cytometry

Plots

Confirm that:

- [ ] The axis labels state the marker and fluorochrome used (e.g. CD4-FITC).
- [ ] The axis scales are clearly visible. Include numbers along axes only for bottom left plot of group (a 'group' is an analysis of identical markers).
- [ ] All plots are contour plots with outliers or pseudocolor plots.
- [x] A numerical value for number of cells or percentage (with statistics) is provided.

Methodology

Sample preparation	Splenic lymphocytes from neonatal interspecific chimeras
Instrument	SH800 (Sony Corp.)
Software	SH800 (Sony Corp.)
Cell population abundance	1,000,000 cells in fluorescent-negative fraction
Gating strategy	Doublets were excluded by FSC-H and FSC-A gatingcell debris were excluded by SSC-A and FSC-A gating. Fluorescence negative fraction was determined using Crlj:WI rat and interspecific chimera.

- [ ] Tick this box to confirm that a figure exemplifying the gating strategy is provided in the Supplementary Information.
